# Antiproliferative and antimetabolic effects behind the anticancer property of fermented wheat germ extract

**DOI:** 10.1186/s12906-016-1138-5

**Published:** 2016-06-01

**Authors:** Christoph Otto, Theresa Hahlbrock, Kilian Eich, Ferdi Karaaslan, Constantin Jürgens, Christoph-Thomas Germer, Armin Wiegering, Ulrike Kämmerer

**Affiliations:** Experimental Surgery, Department of General, Visceral, Vascular, and Pediatric Surgery, University Hospital of Würzburg, Oberdürrbacher Str. 6, D-97080 Würzburg, Germany; Present Address: Spital Bülach, Medizinische Klinik, Spitalstrasse 24, 8180 Bülach, Germany; Present Address: Missionsärztliche Klinik, Fachabteilung Urologie, Salvatorstraße 7, 97074 Würzburg, Germany; Present Address: Klinik für Allgemein-, Viszeral-, Gefäß- und Thoraxchirurgie, Krankenhaus Leopoldina der Stadt Schweinfurt, Gustav-Adolf-Straße 8, 97422 Schweinfurt, Germany; Department of General, Visceral, Vascular and Pediatric Surgery, University Hospital of Würzburg, Oberdürrbacher Str. 6, D-97080 Würzburg, Germany; Department of Biochemistry and Molecular Biology, Theodor-Boveri-Institute, Biocenter, University of Würzburg, D-97070 Würzburg, Germany; Department of Obstetrics and Gynaecology, University Hospital of Würzburg, Josef-Schneider-Str. 4, D-97080 Würzburg, Germany

**Keywords:** FWGE, Benzoquinone, Cancer cells, Reactive oxygen species, Autophagy, Cytotoxicity, Cytostatic

## Abstract

**Background:**

Fermented wheat germ extract (FWGE) sold under the trade name Avemar exhibits anticancer activity in vitro and in vivo. Its mechanisms of action are divided into antiproliferative and antimetabolic effects. Its influcence on cancer cell metabolism needs further investigation. One objective of this study, therefore, was to further elucidate the antimetabolic action of FWGE. The anticancer compound 2,6-dimethoxy-1,4-benzoquinone (DMBQ) is the major bioactive compound in FWGE and is probably responsible for its anticancer activity. The second objective of this study was to compare the antiproliferative properties in vitro of FWGE and the DMBQ compound.

**Methods:**

The IC_50_ values of FWGE were determined for nine human cancer cell lines after 24 h of culture. The DMBQ compound was used at a concentration of 24 μmol/l, which is equal to the molar concentration of DMBQ in FWGE. Cell viability, cell cycle, cellular redox state, glucose consumption, lactic acid production, cellular ATP levels, and the NADH/NAD^+^ ratio were measured.

**Results:**

The mean IC_50_ value of FWGE for the nine human cancer cell lines tested was 10 mg/ml. Both FWGE (10 mg/ml) and the DMBQ compound (24 μmol/l) induced massive cell damage within 24 h after starting treatment, with changes in the cellular redox state secondary to formation of intracellular reactive oxygen species. Unlike the DMBQ compound, which was only cytotoxic, FWGE exhibited cytostatic and growth delay effects in addition to cytotoxicity. Both cytostatic and growth delay effects were linked to impaired glucose utilization which influenced the cell cycle, cellular ATP levels, and the NADH/NAD^+^ ratio. The growth delay effect in response to FWGE treatment led to induction of autophagy.

**Conclusions:**

FWGE and the DMBQ compound both induced oxidative stress-promoted cytotoxicity. In addition, FWGE exhibited cytostatic and growth delay effects associated with impaired glucose utilization which led to autophagy, a possible previously unknown mechanism behind the influence of FWGE on cancer cell metabolism.

**Electronic supplementary material:**

The online version of this article (doi:10.1186/s12906-016-1138-5) contains supplementary material, which is available to authorized users.

## Background

Fermented wheat germ extract (FWGE) with the trade name Avemar is a licensed medical nutriment for cancer patients [1]. The anticarcinogenic potential of FWGE has been demonstrated in vitro and in vivo [2, 3] and confirmed in two clinical trials: an open-label cohort trial with 170 colorectal cancer patients [4], and a randomized phase II trial with 46 stage III melanoma patients [5] receiving continuous supplementation of FWGE along with standard treatment. In both trials, FWGE was found to be beneficial in terms of overall and progression-free survival compared to standard therapy alone.

FWGE is produced as an aqueous extract, which contains the antitumor compounds 2,6-dimethoxy-1,4-benzoquinone (DMBQ) and 2-methoxy-benzoquinone in a concentration of approximately 400 μg/g (0.04 %) crude extract [2]. Quinones are cyclic organic compounds containing two carbonyl groups (C = O) linked to the cyclic structure of a conjugated system. Some clinical anti-cancer compounds, e.g. Mitomycin C, Mitoxantran, Doxorubicin, and Daunorubicin are quinone derivatives [6, 7]. In the 1970s, Bachur et al. described intracellular activation of benzoquinones to free radicals that irreversibly damage biomolecules, e.g. nucleic acids and proteins, but the benzoquinone-mediated production of reactive oxygen species represented the primary source of cell damage [8]. In the mid-1980s, the team of Nobel Prize winner Albert Szent-Györgyi examined the electrochemical and cytotoxic properties of benzoquinones: they injected DMBQ into the peritoneal cavity of mice harboring Ehrlich ascites tumor cells and found a complete elimination of tumor cells [9].

Reactive oxygen species (ROS) represent a broad range of chemically distinct reactive species of radicals with a single unpaired electron, including the superoxide anion radical (O_2_^−^) and the hydroxyl radical (OH^−^), as well as non-radical ROS such as hydrogen peroxide (H_2_O_2_). A marked increase in intracellular ROS can cause oxidative stress with irreversible cell damage [10]. Mammalian cells contain different types of intracellular non-enzymatic and enzymatic antioxidants, e.g. the tripeptide glutathione [11], catalase, and DT-diaphorase that protect them from unwanted oxidative damage [12]. DT-diaphorase plays an important role in protecting cells against endogenous and exogenous quinones [13].

FWGE influences the metabolism of cancer cells [14] which utilize glucose in a way distinct from normal cells [15]. A better understanding of how FWGE influences cancer cell metabolism could improve our understanding of its anticancer activity. The first objective of the present study was to further elucidate the antimetabolic properties of FWGE in cancer cells. The anticancer compound DMBQ appears to be the bioactive molecule in FWGE responsible for its antiproliferative and antimetabolic properties [2]. This assumption has not yet been confirmed experimentally. The second objective of this study, therefore, was to compare the antiproliferative properties of FWGE and the DMBQ compound (at a concentration equal to that in FWGE) in nine human cancer cell lines.

## Methods

### Cell lines

Human malignant cell lines (Table 1) were routinely cultured in RPMI 1640 medium at 37 °C and 5 % CO_2_ supplemented with 10 % (v/v) heat-inactivated fetal calf serum (FCS), 100 U/ml penicillin, 100 μg/ml streptomycin, 2 mmol/l glutamine, 50 mmol/l mercaptoethanol, and 1 % non-essential amino acids in final concentrations (Invitrogen Life Technologies GmbH, Karlsruhe, Germany).

**Table 1:** IC_50_ values of FWGE for different human cancer cell lines. IC_50_ values in mg/ml are shown as mean ± standard deviation (SD) of three independent experiments, each performed in triplicate and calculated as described [21]. Cancer cells were treated for 24 h with different concentrations of FWGE (0.1, 1.0, 10, 50 mg/ml) in medium with 10 % (v/v) fetal calf serum (FCS) and cell viability was determined by crystal violet (CV)

Cell line	Tissue	Mean	SD
MDA-MB-468^a^	Adenocarcinoma of the breast [33]	3.8	1.77
ASPC-1^b^	Adenocarcinoma of the pancreas [34]	4.0	0.37
BxPC-3^b^	Adenocarcinoma of the pancreas [34]	4.4	0.66
MDA-MB-231^a^	Adenocarcinoma of the breast [33]	5.5	0.08
23132/87^b^	Adenocarcinoma of the stomach [35]	7.9	2.05
HT-29^a^	Adenocarcinoma of the colon [36]	10.9	6.36
BT-20^c^	Adenocarcinoma of the breast [37]	13.3	7.41
HRT-18^b^	Adenocarcinoma of the colon [38]	15.8	7.83
MCF-7^a^	Invasive breast ductal carcinoma [37]	19.3	17.66

Cell sources: ^a^CLS: Cell Lines Services GmbH, Eppelheim, Germany (www.clsgmbh.de); ^b^IOP: Institute of Pathology, University of Würzburg, Germany. ^c^DOG: Department of Obstetrics and Gynaecology, University of Würzburg Hospital, Germany

### Drugs and chemicals

FWGE powder from Biropharma, Hungary, was used. For each experiment, a fresh stock solution containing 100 mg/ml FWGE was prepared with RPMI 1640 medium and passed through a 0.2 μm filter. FWG contains benzoquinones in a concentration of approx. 400 μg/g (0.04 %) crude extract as described previously [2]. The antitumor compound DMBQ (molecular weight: 168 g/mol) at 97 % purity (Sigma-Aldrich, Germany) was used in the same molar concentration of 24 μmol/l as described for FWGE (Avemar) [2]. The DMBQ concentration for 10 mg/ml FWGE (=24 μmol/l) was calculated as follows: [10 mg/ml (10 g/l) x 0.04 %]/168 g/mol. For each experiment, a fresh stock solution of 0.1 mg/ml (595 μmol/l) DMBQ was set-up in RPMI 1640 medium at 37 °C for 10 min to improve solubility. After passing the stock solution through a 0.2 μm filter, no DMBQ sediment was observed. A fresh and sterile stock solution of 2.4 mmol/l ascorbic acid in RPMI 1640 medium was prepared as described [16].

### Measurement of FWGE effects on cell growth

Cells (1.5×10^4^) were seeded in 200 μl culture medium per well into 96-well flat-bottom tissue plates (Greiner bio-one, Germany). The next day, cells were incubated for 24 h with fresh culture medium containing the final concentrations of 0.1, 1.0, 10, 50 mg/ml FWGE according to published data [17–19]. The tissue plates were cultured at 37 °C in a humidified atmosphere of 5 % CO_2_ in air and cellular viability was determined by crystal violet (CV) staining as described previously [20]. The absorbance (optical density, OD) was measured with a microplate reader (MRX, Dynatech Laboratories) at a wave length of 570 nm which is directly proportional to the number of viable cells. Dose–response curves were used to calculate IC_50_ values as described [21]. After a 24 h-exposure with 10 mg/ml FWGE, the following three antiproliferative effects (which influence the number of viable cells) were observed: cytotoxic, cytostatic, and growth delay. By definition, a cytotoxic effect is a reduction in initial viable cell count >15 %, a cytostatic effect a change in initial viable cell count ±15 %, and delayed growth effect an increase in initial viable cell count >15 %.

### Determination of cellular ATP content and NADH/NAD^+^ ratio

Cellular ATP content was determined with the Colorimetric/Fluorometric Assay Kit (K354-100) from BioVision, USA according to the manufacturer’s instructions. ATP content was given in pg/10^6^ cells for 24 h. The NADH/NAD^+^ ratio, determined with the quantitation colorimetric kit (K337-100) from BioVision, was given for 10^4^ cells.

### Measuring cellular redox state

The OxiSelect™ lntracellular ROS Assay Kit (Gell Biolabs, USA; STA-342) is a cell-based assay for measuring the activity of hydroxyl, peroxyl, and other reactive oxygen species within a cell. The assay employs the redox-sensitive fluorogenic dye DCFH-DA, which diffuses into cells and is deacetylcated by cellular esterases into the non-fluorescent DCFH. ln the presence of ROS, DCFH is rapidly oxidized to highly fluorescent DCF. Fluorescence was quantified 12 h and 24 h after incubation with FWGE and DMBQ on a standard fluorescence plate reader at 480/530 nm. Results are presented as relative fluorescence units normalized for 10^4^ cells.

### Glucose consumption and lactic acid production

Cells (1.5×10^4^) were seeded in 200 μl culture medium per well into 96-well flat-bottom tissue plates (Greiner bio-one). After 24 h, 48 h, and 72 h of culture, cell-free supernatant was analyzed for glucose consumption and lactic acid production by the central laboratory of the University Hospital of Würzburg using the Cobas 8000 modular analyzer series (Roche Diagnostics, Germany). Glucose consumption was calculated from the difference between glucose concentration in cell­free control medium and glucose concentration remaining in the supernatant of cell cultures after incubation. Lactic acid production was calculated from the difference between lactic acid concentrations in the supernatant of cell cultures before and after incubation. Results were correlated to the cell count and displayed as consumption/production per 10^4^ cells.

### Western blotting

Western blotting was performed as described earlier [22, 23]. ln brief, 1 × 10^6^ cells each were lysed in pre-cooled RIPA buffer (Pierce, USA) containing phosphatase and proteinase inhibitors and 2.5 mmol/l dithiothreitol (Sigma-Aldrich). Equal amounts of proteins (30 μg) were loaded on a 10 % polyacrylamide gel (SDS-PAGE), electrophoresed, and then blotted by semi-dry transfer onto a nitrocellulose membrane (Schleicher & Schuell, Germany). After a blocking step with 5 % non-fat milk (Merck, Germany), membranes were incubated with either a rabbit anti-DT diaphorase primary antibody (NQ01, N5288, Sigma-Aldrich; diluted 1:2,000) or a rabbit anti-LC3-I/-II primary antibody (AHP2167T; AbD Serotec GmbH, Germany; diluted 1:1,000). After washing with phosphate buffered saline (PBS), membranes were incubated with a horseradish peroxidase­conjugated goat anti-rabbit secondary antibody (KPL, USA; diluted 1:10,000) for 60 min at room temperature. A monoclonal mouse anti-β-actin primary antibody (Abcam, UK; diluted 1:10,000) was used as loading control and visualized with the goat anti-mouse secondary antibody (KPL, diluted 1: 10,000). lmmunoblots were visualized by enhanced chemiluminescence western blotting substrate (Pierce, Thermo Scientific, USA) with subsequent exposure on an X-ray film (Fuji Super RX medical X-ray films; Fuji, Germany) for 30 s.

### Cell cycle analysis

For cell cycle analysis, 1×10^6^ cells were fixed in suspension with 70 % ice-cold ethanol (−20 °C) and incubated for 2 h at 4 °C. Subsequently, ice-cold PBS was added and the cells were pelleted at 250 xg for 6 min at 4 °C. Cell pellets were resuspended with PBS. RNase (lnvitrogen GmbH, Germnay) was added for a final concentration of 50 μg/ml and incubated at 37 °C for 30 min in the dark. Then propidium iodide (Sigma-Aldrich; stock solution: 1 mg/ml, final concentration: 50 μg/ml) was added. After 5 min of incubation, the cells were measured for their DNA amount with a FACScan (Becton Dickinson, Germany). Data (10,000 events per acquisition) were recorded with BD CellQuest™ Pro software (Version 5.1.1) and data were analyzed with WinMDI software (Version 2.9).

### Statistical analysis

The experiments were performed at least three times with triplicate samples. The means were compared using analysis of variance (ANOVA) plus Bonferroni’s *t*-test. A *P*-value of <0.05 was considered to indicate a statistically significant result.

## Results

### Antiproliferative properties of FWGE

The mean IC_50_ value of FWGE determined for nine cancer cell lines tested after 24 h of culture was 10 mg/ml (range from 3.8 to 19.3 mg/ml) (Table 1). The pancreatic cancer cell lines BxPC-3 and ASPC-1 and breast cancer cell lines MDA-MB-468 and MDA-MB-231 were extremely sensitive to FWGE with IC_50_ values ranging from 3.8 ± 1.77 to 5.5 ± 0.08 mg/ml. FWGE had less effect on the viability of MCF-7 cells (19.3 ± 17.66 mg/ml), which were the most insensitive cells to FWGE tested in this study. The calculation of Kendall's tau-b rank correlation coefficient displayed a strong correlation between the EC_50_ values of the cell lines tested and the antiproliferative properties of FWGE (*r* = 0.545, *P* < 0.05). In cell lines with IC_50_ < 6.0 mg/ml, FWGE usually triggered cytotoxic effects, in cell lines with IC_50_ > 6.0 mg/ml, FWGE usually triggered cytostatic effects.

**Fig. 1:**
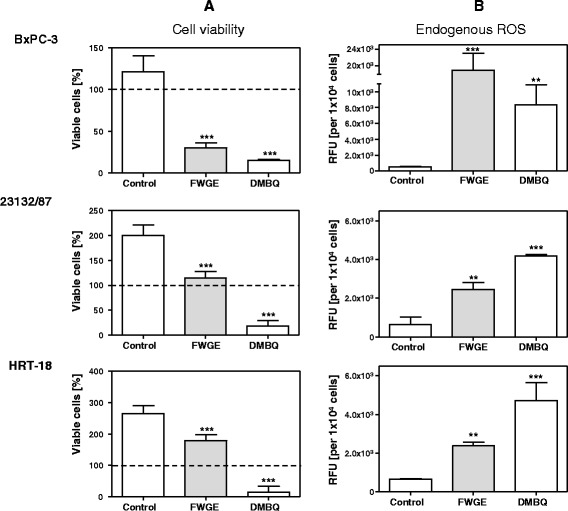
Antiproliferative properties of FWGE and DMBQ. The effects of FWGE (mean IC_50_: 10 mg/ml) and DMBQ (24 μmol/l; equal to the DMBQ concentration in FWGE) on cancer cell viability (**a**). Intracellular DCF fluorescence signals indicating intracellular ROS formation after 12 h (BxPC-3 cells) and 24 h (23132/87 and HRT-18 cells) of culture (**b**). The dashed line indicates the relative initial cell count at the start of treatment. For this, the seeded cells were stained with crystal violet directly after their adherence and the absorbance was normalized to 100 %. By definition, a cytotoxic effect was a reduction in initial viable cell count >15 %, a cytostatic effect a change in initial cell count of ±15 %, and a delayed growth effect an increase in the initial cell count >15 %. Ascorbic acid (2.4 mmol/l) was used to activate DMBQ [16]. Ascorbic acid had no influence on cancer cell viability or FWGE (not shown). Results are shown as mean ± standard deviation and representative for at least three independent experiments performed in triplicate. **P < 0.01, ***P < 0.001 in comparison to untreated control cells. RFU, relative fluorescence units

Characterization of the antiproliferative effect of the mean IC_50_ value of 10 mg/ml FWGE after 24 h treatment of the nine cancer cell lines revealed a cytotoxic effect in four cell lines (ASPC-1, BxPC3, MDA-MB-231, MDA-MB-468), a cytostatic effect in four cell lines (23132/87, BT-20, HT-29, MCF-7) and a growth delay effect in HRT-18 cells (Additional file 1: Figure S1 and Fig. 1a). FWGE at a concentration of 1 mg/ml had no effect on cell viability and 50 mg/ml was cytotoxic for all nine cell lines after a 24 h treatment (not shown). A concentration of 10 mg/ml FWGE has been reported to induce biological responses in normal peripheral blood lymphocytes in vitro [18]. Therefore, we did not use concentrations >10 mg/ml FWGE to analyze its antiproliferative effects.

### Antiproliferative properties of FWGE and DMBQ

One cell line each was selected to investigate the three antiproliferative properties of FWGE: BxPC-3 cells (cytotoxic effect), 23132/87 cells (cytostatic effect) and HRT-18 cells (growth delay effect). The DMBQ compound was used in a concentration of 24 μmol/l, which is equal to its concentration in FWGE. At this concentration, the DMBQ compound induced strong cytotoxicity in all nine cancer cell lines tested after 24 h of treatment. Representative results for the three cell lines BxPC-3, 23132/87 and HRT-18 are shown in Fig. 1a, results for other cell lines in the Additional file 1: Figure S1. BxPC3 cells reacted very sensitively to treatment with FWGE and DMBQ. The cytotoxicity induced by DMBQ-mediated ROS is well known [8, 9]. We found that the massive cell damage induced by the DMBQ compound and FWGE was linked to aberrant levels of intracellular DCF fluorescence. This shift in the cellular redox state was caused by intracellular ROS (Fig. 1b) and was confirmed by experiments with exogenous glutathione (Additional file 2: Figure S2). Exogenous glutathione (GSH) protected BxPC-3 cells against DMBQ compound/FWGE-induced cell damage, confirming the oxidative-type cytotoxicity of DMBQ and FWGE. The strong cytotoxic effect of FWGE and the DMBQ compound in BxPC-3 cells was linked to a loss of the enzyme DT-diaphorase (Additional file 3: Figure S3) which protects cells against benzoquinone-induced oxidative stress. The FWGE-induced cytostatic and growth delay effects in 23132/87 and HRT-18 cells were linked to moderate ROS levels (Fig. 1b). In contrast to its cytotoxic effect, FWGE’s cytostatic and growth delay effects were not influenced by GSH (Additional file 2: Figure S2).

### Metabolic effects of FWGE

**Fig. 2:**
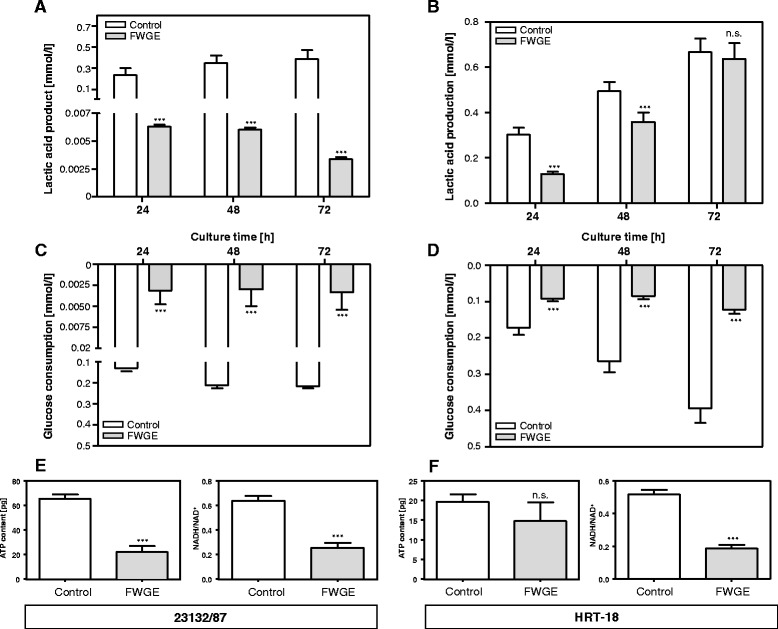
FWGE-influenced cancer cell metabolism. Lactic acid production (mmol/l for 10^4^ cells) and glucose consumption (mmol/l for 10^4^ cells) by 23132/87 cells (**a**, **c**) and HRT-18 cells (**b**, **d**) with and without FWGE treatment for the incubation times indicated. Cellular ATP content (pg/10^6^ cells) and the NADH/NAD^+^ ratio in 23132/87 cells (**e**) and HRT-18 cells (**f**) with and without FWGE treatment after 24 h of culture. Results are shown as mean ± standard deviation for three independent experiments.***P < 0.001 to FWGE-untreated cells

The cytostatic and growth delay effects of FWGE in 23132/87 and HRT-18 cells indicate that it influences cell metabolism and function. We found that the FWGE-induced cytostatic effect in 23132/87 cells is based on cell cycle arrest (Additional file 4: Figure S4). In addition, we found that the FWGE-induced cytostatic effect in 23132/87 cells was linked to impaired glucose consumption and significantly reduced production of lactic acid (*P* < 0.01, Fig. 2a,c). In HRT-18 cells, the growth delay effect of FWGE was also linked to impaired glucose consumption and the cells produced less lactic acid (Fig. 2b,d). However, in comparison to untreated HRT-18 cells, it was obvious that FWGE-treated HRT-18 cells produced more lactic acid than expected based on overall glucose consumption after 48 and 72 h of culture (Fig. 2b,d). At 72 h of culture, FWGE-treated HRT-18 cells consumed four-fold less glucose than FWGE-untreated cells but produced the same levels of lactic acid, indicating that HRT-18 cells are able to shift their metabolism to other energy sources than glucose linked with production of lactic acid, e.g. glutamine via glutaminolysis.

Impaired glucose consumption can affect cellular energy sources. We therefore measured cellular ATP and NADH levels in 23132/87 and HRT-18 cells. FWGE-treated 23132/87 cells exhibited a 66 % depletion in cellular ATP and a 60 % decrease in the NADH/NAD^+^ ratio (Fig. 2e). In HRT-18 cells, FWGE also decreased the NADH/NAD^+^ ratio by 64 % (Fig. 2f) with unchanged cellular ATP content. The decrease in the NADH/NAD^+^ ratio, reflecting an increase in the amount of (non-reduced) NAD^+^, indicates that FWGE influenced the reduction of the NAD^+^ pool in these cells.

### FWGE-induced autophagy

**Fig. 3:**
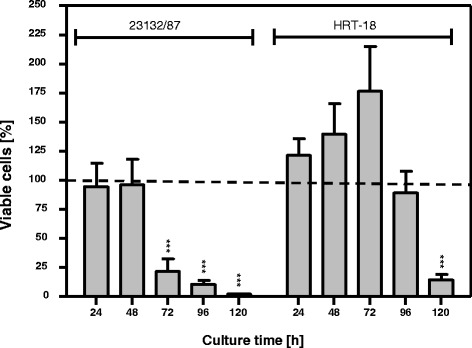
Viability of HRT-18 and 23132/87 cells continuously cultured with FWGE. Incubation of 23132/87 and HRT-18 cells with 10 mg/ml FWGE was cytotoxic after 72 h of continuous culture for 23132/87 cells and after 120 h for HRT-18 cells. Cells were incubated in medium with 10 % (v/v) fetal calf serum and cell viability was determined by crystal violet staining at the culture times indicated. The dashed line indicates the relative initial cell count at the start of treatment. For this, the seeded cells were stained with crystal violet directly after their adherence and the absorbance was normalized to 100 %. By definition, a cytotoxic effect was a reduction in initial viable cell count >15 %, a cytostatic effect a change in initial cell count ±15 % and a delayed growth effect an increase in the initial cell count >15 %. Results are shown as mean ± standard deviation for three independent experiments, each performed in triplicate for each time point. ***P < 0.001 in comparison to 24 h

**Fig. 4:**
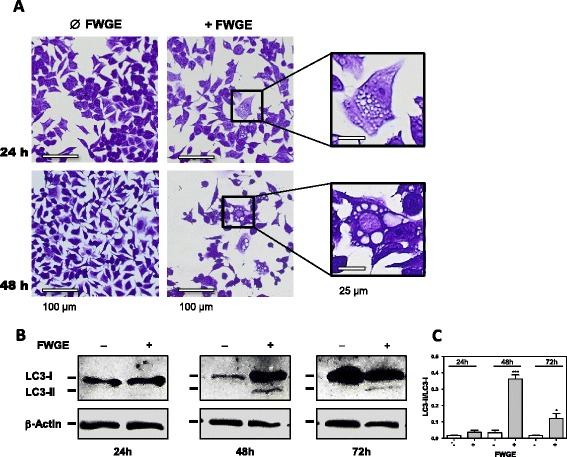
FWGE-induced autophagic activity in the colon carcinoma cell line HRT-18. FWGE-treated HRT-18 cells showing intracellular vacuoles 24 h after start of incubation. The vacuoles increased in size with increasing incubation time (**a**). Presence of endogenous LC3-II in HRT-18 cells with and without FWGE treatment at different incubation times (**b**). LC3-I (approximately 17 kDa) is the cytosolic form of LC3, which is converted into the active, membrane-bound form LC3-II (approximately 14–15 kDa) during the autophagy process. β-actin was used as a loading control (42 kDa). The shift from LC3-I to LC3-II is evident following FWGE treatment. Western blot results are shown for one trial representative of three independent experiments (**c**). The LC3-II/LC3-I ratio was calculated based on densitometry analysis (ImageJ 1.3.4 s downloaded from the National Institutes of Health (NIH), Bethesda, MD, USA) of LC3-I and LC3-II bands for three independent experiments (***P < 0.001, *P < 0.05 in comparison to FWEG-untreated cells)

The unchanged ATP content in FWGE-treated HRT-18 cells (Fig. 2f) was linked to growth delay (Fig. 1) with prolonged cell survival in continuous culture with FWGE (Fig. 3). Microscopic analysis of FWGE-treated HRT-18 cells demonstrated the formation of intracellular vacuoles, first observed 24 h after starting incubation, which increased in size with increasing incubation time (Fig. 4). To determine whether the FWGE-induced formation of intracellular vacuoles was a characteristic sign of autophagy, a pro-survival self-degradation process under metabolic stress, we analyzed the cells for the presence of the autophagy marker LC3-II. With an anti-LC3 antibody, we confirmed the presence of LC3-II exclusively in FWGE-treated HRT-18 cells (Fig. 4). This indicates an accumulation of HRT-18 cells within the cell population demonstrating autophagic activity during incubation with FWGE.

## Discussion

ln this study we analyzed the antiproliferative and antimetabolic effects of fermented wheat germ (FWGE) sold under the trade name Avemar. Because FWGE contains the anticancer compound DMBQ, which is thought to be responsible for the antiproliferative and antimetabolic properties of FWGE [2], we investigated whether FWGE and DMBQ exhibit similar antiproliferative effects. For this purpose, the DMBQ compound was used in a molar concentration of 24 μmol/l equal to its concentration in FWGE [2, 3]. FWGE and DMBQ were tested on nine human cancer cell lines derived from different cancer types (Table 1). ln addition, the effect of Avemar was also tested on normal human dermal fibroblasts (PromoCell, Germany) with an IC_50_ value of 35.5 ± 20.5 mg/ml (not shown). 24 μmol/l of the DMBQ compound was found to be cytotoxic for all cancer cell lines tested within 24 h (Additional file 1: Figure S1), whereas 10 mg/ml FWGE (mean IC_50_ value) exhibited additional cytostatic and growth delay effects. In this context, it is worth mentioning that 10 mg/ml FWGE in continuous culture with cancer cells was cytotoxic (Fig. 3), as was 50 mg/ml FWGE for 24 h (not shown). The treatment of cells with 10 mg/ml FWGE for 24 h allowed us to investigate the antimetabolic mechanisms of FWGE in detail.

DMBQ-mediated ROS-induced cytotoxicity is well known [8, 9, 24, 25]. We found that DMBQ-induced cell damage was linked to increased intracellular DCF fluorescence. A comparable increase in DCF fluorescence was also found in BXPC-3 cells incubated with FWGE and indicates the production of intracellular ROS. Detection of ROS based on DCF fluorescence is the most widely used assay but various caveats apply [26]. Our present findings provide further evidence for DMBQ/FWGE-induced ROS production, showing that exogenous glutathione (GSH) protected BxPC3 cells against DMBQ/FWGE-induced cell damage. Cellular glutathione levels are maintained by *de novo* synthesis, reduction of glutathione disulfide, and glutathione uptake from exogenous sources [11], underlining the role of glutathione as a free radical scavenger in cell cultures as described elsewhere [25]. In addition, the thiolic antioxidant N-acetylcysteine and catalase both displayed protective effects and prevented DMBQ/FWGE-induced cell damage (not shown). In contrast to its cytotoxic effect, the cytostatic and growth delay effects of FWGE appear to be independent of oxidative stress and glutathione had no observable effect on cell viability.

A review of the literature shows that intracellular flavoenzymes play an important role in quinone bioactivation [25]. In addition, activation of DMBQ outside the cell with ROS-induced lipid peroxidation is described as a possible mechanism for quinone cytotoxicity [25, 27]. The barrier function of the plasma membrane is lost and DMBQ diffuses through the open plasma membrane into the cytoplasm with intracellular ROS production. With the exception of BxPC3 and ASPC-1 cells, DMBQ showed a need for ascorbic acid in order to induce DMBQ-mediated ROS production. Ascorbic acid acts as electron donor and reduces DMBQ to semiquinone radicals [9]. It can be transported across the plasma membrane into the cell via the sodium-dependent vitamin C transporter or, in its oxidized form, via glucose transporter, including the ubiquitously expressed Glut1 [28]. In contrast to DMBQ, the antiproliferative effect of FWGE was not influenced by ascorbic acid (not shown).

Some of the mechanisms of action for FWGE can be classified as metabolic effects [14]. For example, FWGE prevents glucose uptake into cells and inhibits key enzymes of glycolysis such as hexokinase and lactate dehydrogenase [17, 18]. Under sufficient oxygenation, normal cells direct glucose predominantly to mitochondrial oxidative phosphorylation to generate ATP, while cancer cells often exhibit nonoxidative glucose utilization, which enhances lactic acid production by lactate dehydrogenase (LDH). The reaction of LDH leads to the oxidization of NADH to NAD^+^, necessary to support glycolytic flux [15]. The exact role and regulation of a hyperactivated glycolytic pathway in cancer cells, termed aerobic glycolysis or the Warburg effect, is still not fully understood. Its major benefit to cancer cells is rapid ATP production and increased supply with anabolic substrates [29]. To determine FWGE-induced alterations in cancer cell metabolism, we measured glucose consumption and generation of lactic acid during cell culture. FWGE impaired glucose consumption of 23132/87 cells and HRT-18 cells caused a low NADH/NAD^+^ ratio, an indication of decreased glucose flux through glycolysis. In contrast to 23132/87 cells, FWGE-treated HRT-18 cells formed more lactic acid than would be expected from the low glucose consumption. An alternative pathway for the generation of lactic acid independent of glucose utilization is glutaminolysis. This pathway is involved in the conversion of cytosolic malic acid into pyruvic acid by malic enzymes [30], where excess pyruvic acid is then depleted by LDH. The impact of glutamine on formation of lactic acid independent of glycolysis by FWGE-treated HRT-18 cells was not an object of this study and will be addressed in further studies.

FWGE-treated HRT-18 cells exhibited autophagic activity as demonstrated by the presence of the autophagy marker LC3-II [31]. Autophagy is a self-degradation process which is proposed to have a pro-survival effect for cancer cells under metabolic stress by shifting the energy production from glycolysis towards degradation of unneeded proteins and fatty acids to feed the citric acid cycle for generating ATP [32]. In this context, we found unchanged ATP levels in FWGE-treated HRT-18 cells, indicating that they are able to compensate the impaired glucose utilization during incubation with FWGE and maintain glycolysis-independent ATP production. In addition, HRT-18 cells had prolonged cell survival during continuous culture with FWGE in comparison to 23132/87 cells, which did not exhibit autophagy (Fig. 3). Taken together, the antiproliferative properties of FWGE display a complex interaction with cancer cell metabolism.

## Conclusions

The antiproliferative properties of FWGE are complex and differ in some respects from those of the DMBQ compound. This may explain why there is to date no evidence of toxic side effects from FWGE in clinical trials in contrast to clinically applied quinone compounds. In addition to its cytotoxic effect, FWGE also has cytostatic and growth delay effects at a concentration of 10 mg/ml after 24 h of incubation, while 24 μmol/l of the DMBQ compound (equal to the DMBQ concentration in FWGE) was uniformly cytotoxic for all cancer cell lines we tested. The oxidative cell damage potential of activated DMBQ was confirmed by aberrant intracellular DCF fluorescence, indicating increased levels of intracellular ROS. A marked increase of ROS was also found to underlie the cytotoxic effect of FWGE. Moderate levels of intracellular ROS were found to underlie the cytostatic and growth delay effects of FWGE which were linked to impaired glucose utilization and induction of autophagy, a previously unknown mechanism of FWGE for targeting cancer cell metabolism.

## Abbreviations

ATP, adenosine triphosphate; CV, crystal violet; DCFH-DA, 2′,7′-dichlorofluorescin diacetate; DMBQ, 2,6-dimethoxy-1,4-benzoquinone; FWGE, fermented wheat germ extract; IC_50_, half maximal inhibitory concentration; NAD, nicotinamide adenine dinucleotide; PBS, phosphate buffered saline; RFU, relative fluorescence units; ROS, reactive oxygen species.

## Additional files

Additional file 1: Figure S1. Antiproliferative properties of FWGE and DMBQ on cancer cells. 10 mg/ml FWGE exhibited cytotoxic (**a**) and cytostatic (**b**) effects after 24 h of culture. The growth delay effect in HRT-18 cells is shown in Fig. 1. Representative figures of crystal violet stained viable cancer cells treated with FWGE and DMBQ after 24 h of culture (**c**). DMBQ displayed a strong cytotoxic effect in all cancer cell lines. The dashed line indicates the relative initial cell count at the start of treatment. For this, the seeded cells were stained with crystal violet directly after their adherence and the absorbance was normalized to 100 %. By definition, a cytotoxic effect was a reduction in initial viable cell count >15 %, a cytostatic effect a change in initial cell count ±15 % and a delayed growth effect an increase in the initial cell count >15 %. Ascorbic acid (2.4 mmol/l) was used to activate DMBQ [16] and had no influence on cell viability or the effect of FWGE (not shown). Results are shown as mean ± standard error of mean (S.E.M.) and representative for at least three independent experiments performed in triplicate. Magnification: 80x. **P* < 0.05, ***P* < 0.01, ****P* < 0.001 in comparison to untreated control cells. (PDF 216 kb) Additional file 2: Figure S2. The protective effect of exogenous glutathione on the viability of cancer cells treated with FWGE/DMBQ. The protective effects of exogenous glutathione (GSH) protected against DMBQ and FWGE-induced cytotoxicity in BxPC-3, 23132/87, and HRT-18 cells. GSH did not influence FWGE-induced cytostatic and growth delay effects. Cancer cells were treated with FWGE (10 mg/ml) or DMBQ (24 μmol/l) with (+) and without (−) GSH for 24 h. The GSH concentration (3.6 mmol/l) used was optimal as determined in previous studies. The dashed line indicates the relative initial cell count at the start of treatment. For this, the seeded cells were stained with crystal violet directly after their adherence and the absorbance was normalized to 100 %. Results present the mean (±S.E.M.) of three independent experiments, each performed in triplicate. Cancer cells were cultured in RPMI 1640 medium with 10 % (v/v) fetal calf serum (FCS). ***P* < 0.01 in comparison to untreated control cells, n.s. = not significant. (PDF 15 kb) Additional file 3: Figure S3. Demonstration of the presence of DT-diaphorase in cancer cells and fibroblasts. ASPC-1 and BxPC-3 cells exhibited a complete loss of the enzyme DT-diaphorase (NAD(P)H:quinone oxidoreductase, NQO1), which protects cells particularly against benzoquinone-induced oxidative stress and can explain the sensitivity of BxPC-3 and ASPC-1 cells (not shown) to incubation with FWGE and DMBQ. Whole cell extracts of ASPC-1, BxPC-3, 23132/87, HRT-18 cells and normal human dermal fibroblasts (NHDF from PromoCell, Germany) were separated on SDS-PAGE and probed with rabbit anti-DT-diaphorase antibody, which detects a protein band of 28 kDa. A monoclonal mouse anti-β-actin primary antibody was used as loading control (42 kDa). (PDF 26 kb) Additional file 4: Figure S4. Cell cycle analysis of FWGE-treated and untreated cells. The cell cycle was analyzed 24 h after start of incubation with FWGE (10 mg/ml). Isolated nuclei were stained with propidium iodide (PI) and then subjected to flow cytometry analysis for their DNA content. FACS profiles for 23132/87 cells (**a**) and HRT-18 cells (**b**). Results are shown as mean ± S.E.M. from three different experiments. The bar graph shows the percentages of cells in G1, S, and G2/M. **P* < 0.05, n.s., not significant. (PDF 121 kb)
