# Supplementary figures and images for: Antiproliferative and antimetabolic effects behind the anticancer property of fermented wheat germ extract
Source: BMC Complement Altern Med. 2016 Jun 1;16:160. doi: 10.1186/s12906-016-1138-5 (PMC4888675; doi:10.1186/s12906-016-1138-5)

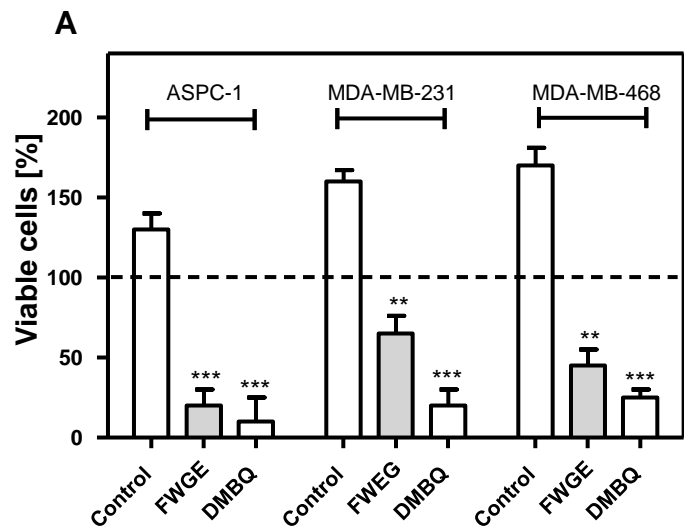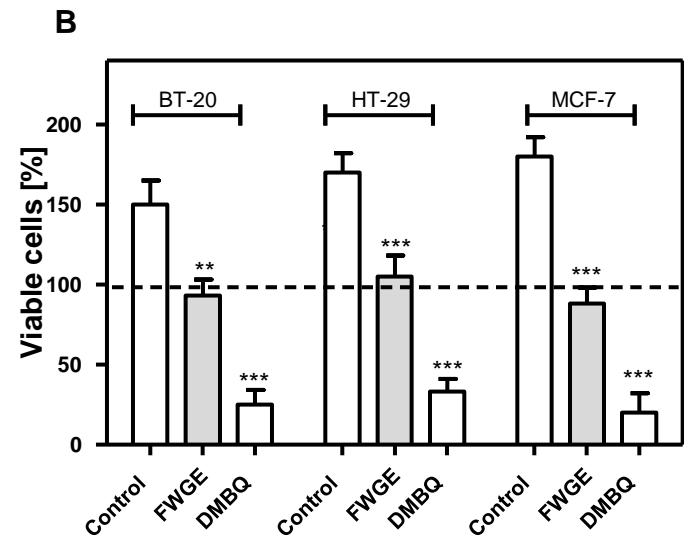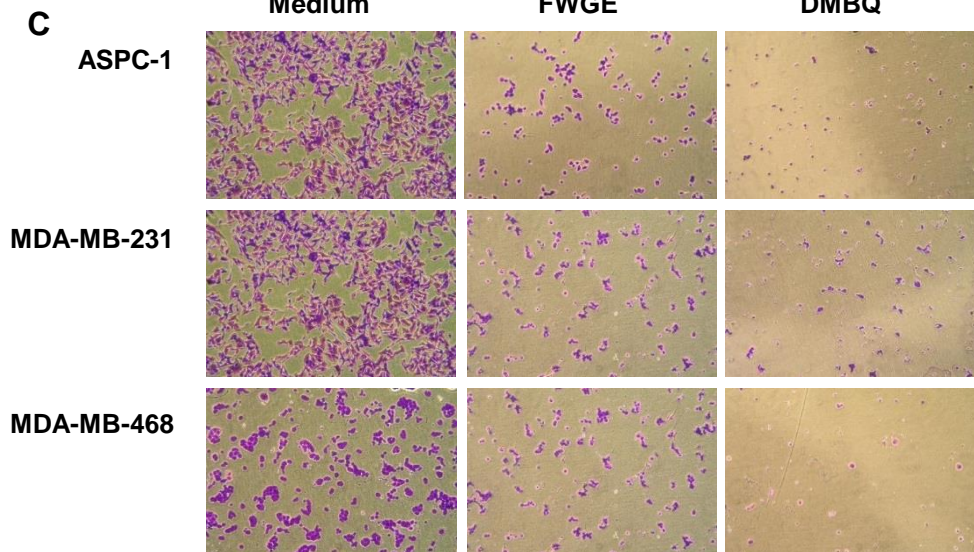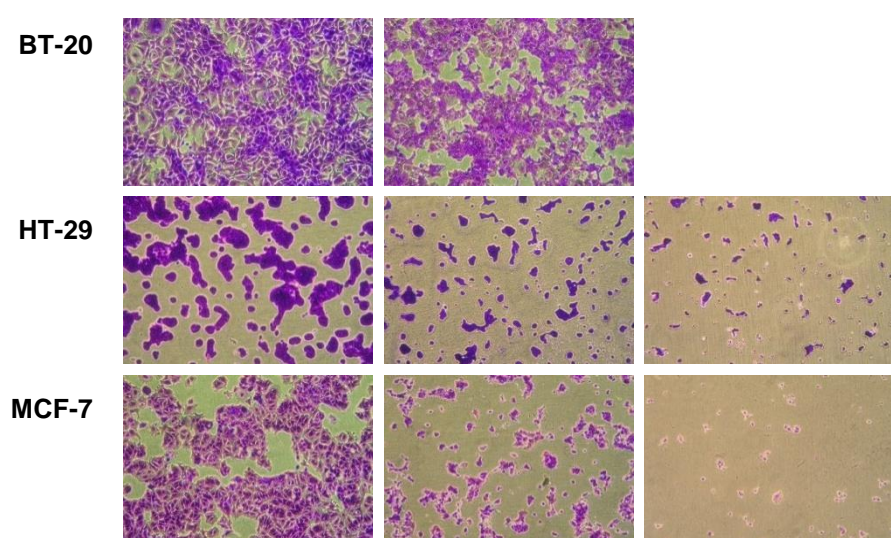

Supplement: Additional file 1: Figure S1. — Antiproliferative properties of FWGE and DMBQ on cancer cells. 10 mg/ml FWGE exhibited cytotoxic (a) and cytostatic (b) effects after 24 h of culture. The growth delay effect in HRT-18 cells is shown in Fig. 1. Representative figures of crystal violet stained viable cancer cells treated with FWGE and DMBQ after 24 h of culture (c). DMBQ displayed a strong cytotoxic effect in all cancer cell lines. The dashed line indicates the relative initial cell count at the start of treatment. For this, the seeded cells were stained with crystal violet directly after their adherence and the absorbance was normalized to 100 %. By definition, a cytotoxic effect was a reduction in initial viable cell count >15 %, a cytostatic effect a change in initial cell count ±15 % and a delayed growth effect an increase in the initial cell count >15 %. Ascorbic acid (2.4 mmol/l) was used to activate DMBQ [16] and had no influence on cell viability or the effect of FWGE (not shown). Results are shown as mean ± standard error of mean (S.E.M.) and representative for at least three independent experiments performed in triplicate. Magnification: 80x. *P < 0.05, **P < 0.01, ***P < 0.001 in comparison to untreated control cells. (PDF 216 kb) [file 12906_2016_1138_MOESM1_ESM.pdf]

BxPC-3

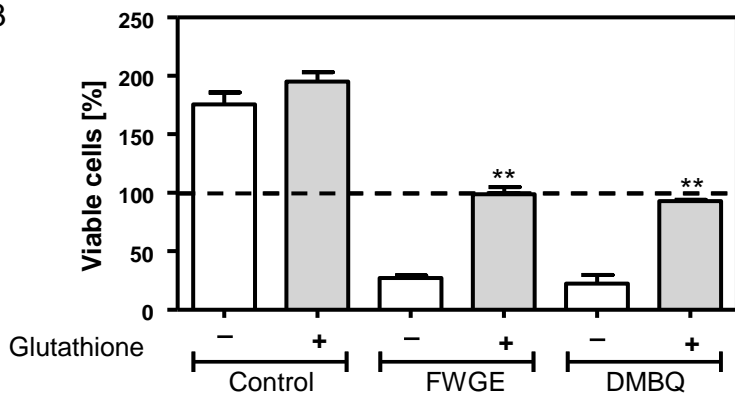

23132/87

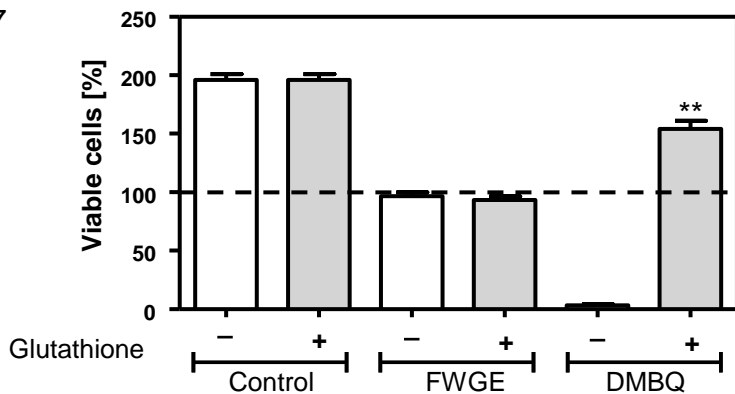

HRT-18

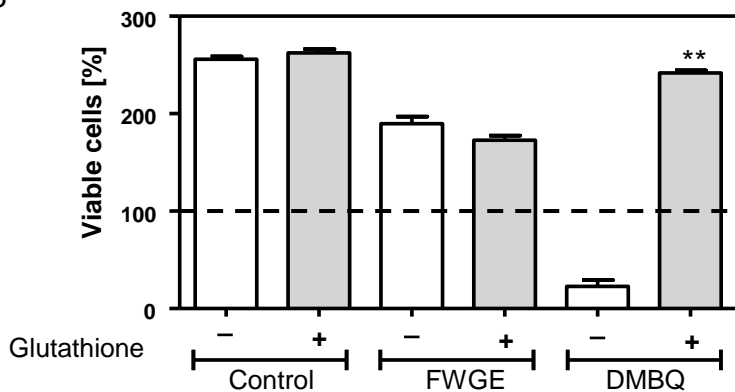

Supplement: Additional file 2: Figure S2. — The protective effect of exogenous glutathione on the viability of cancer cells treated with FWGE/DMBQ. The protective effects of exogenous glutathione (GSH) protected against DMBQ and FWGE-induced cytotoxicity in BxPC-3, 23132/87, and HRT-18 cells. GSH did not influence FWGE-induced cytostatic and growth delay effects. Cancer cells were treated with FWGE (10 mg/ml) or DMBQ (24 μmol/l) with (+) and without (−) GSH for 24 h. The GSH concentration (3.6 mmol/l) used was optimal as determined in previous studies. The dashed line indicates the relative initial cell count at the start of treatment. For this, the seeded cells were stained with crystal violet directly after their adherence and the absorbance was normalized to 100 %. Results present the mean (±S.E.M.) of three independent experiments, each performed in triplicate. Cancer cells were cultured in RPMI 1640 medium with 10 % (v/v) fetal calf serum (FCS). **P < 0.01 in comparison to untreated control cells, n.s. = not significant. (PDF 15 kb) [file 12906_2016_1138_MOESM2_ESM.pdf]

DT-diaphorase

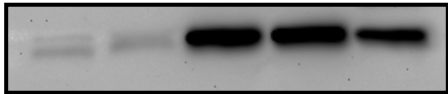

$\beta$ -Actin

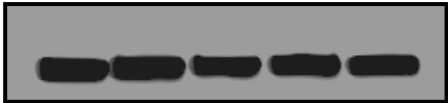

Supplement: Additional file 3: Figure S3. — Demonstration of the presence of DT-diaphorase in cancer cells and fibroblasts. ASPC-1 and BxPC-3 cells exhibited a complete loss of the enzyme DT-diaphorase (NAD(P)H:quinone oxidoreductase, NQO1), which protects cells particularly against benzoquinone-induced oxidative stress and can explain the sensitivity of BxPC-3 and ASPC-1 cells (not shown) to incubation with FWGE and DMBQ. Whole cell extracts of ASPC-1, BxPC-3, 23132/87, HRT-18 cells and normal human dermal fibroblasts (NHDF from PromoCell, Germany) were separated on SDS-PAGE and probed with rabbit anti-DT-diaphorase antibody, which detects a protein band of 28 kDa. A monoclonal mouse anti-β-actin primary antibody was used as loading control (42 kDa). (PDF 26 kb) [file 12906_2016_1138_MOESM3_ESM.pdf]

**A** 23132/87

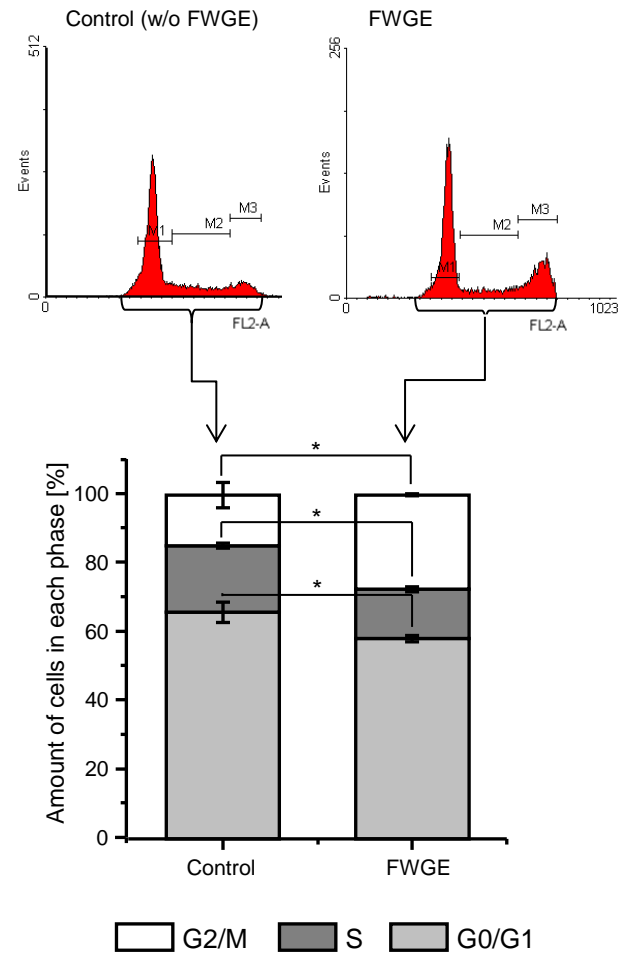

**B** HRT-18

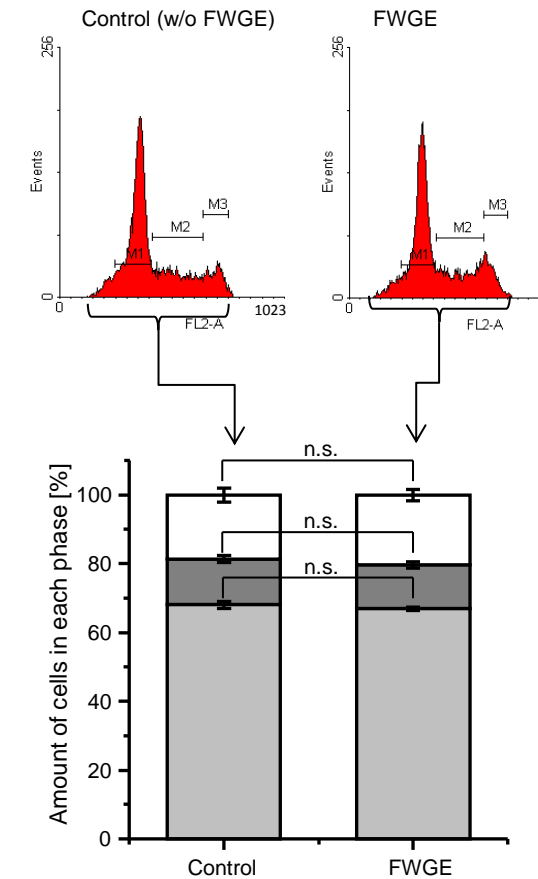

Supplement: Additional file 4: Figure S4. — Cell cycle analysis of FWGE-treated and untreated cells. The cell cycle was analyzed 24 h after start of incubation with FWGE (10 mg/ml). Isolated nuclei were stained with propidium iodide (PI) and then subjected to flow cytometry analysis for their DNA content. FACS profiles for 23132/87 cells (a) and HRT-18 cells (b). Results are shown as mean ± S.E.M. from three different experiments. The bar graph shows the percentages of cells in G1, S, and G2/M. *P < 0.05, n.s., not significant. (PDF 121 kb) [file 12906_2016_1138_MOESM4_ESM.pdf]
